# Age Differences in the Efficiency of Filtering and Ignoring Distraction in Visual Working Memory

**DOI:** 10.3390/brainsci10080556

**Published:** 2020-08-14

**Authors:** Mariana R. Maniglia, Alessandra S. Souza

**Affiliations:** 1 Department of Psychology, Ribeirão Preto School of Philosophy, Science and Literature (FFCLRP), University of São Paulo, Avenida Bandeirantes 3900, Ribeirão Preto/SP 14040-901, Brazil; marianamaniglia@usp.br; 2 Department of Psychology, Cognitive Psychology Unit, University of Zurich, Binzmühlestrasse 14/22, 8050 Zurich, Switzerland

**Keywords:** attention, working memory, aging, filtering, ignoring, precue, retrocue

## Abstract

Healthy aging is associated with decline in the ability to maintain visual information in working memory (WM). We examined whether this decline can be explained by decreases in the ability to filter distraction during encoding or to ignore distraction during memory maintenance. Distraction consisted of irrelevant objects (Exp. 1) or irrelevant features of an object (Exp. 2). In Experiment 1, participants completed a spatial WM task requiring remembering locations on a grid. During encoding or during maintenance, irrelevant distractor positions were presented. In Experiment 2, participants encoded either single-feature (colors or orientations) or multifeature objects (colored triangles) and later reproduced one of these features using a continuous scale. In multifeature blocks, a precue appeared before encoding or a retrocue appeared during memory maintenance indicating with 100% certainty to the to-be-tested feature, thereby enabling filtering and ignoring of the irrelevant (not-cued) feature, respectively. There were no age-related deficits in the efficiency of filtering and ignoring distractor objects (Exp. 1) and of filtering irrelevant features (Exp. 2). Both younger and older adults could not ignore irrelevant features when cued with a retrocue. Overall, our results provide no evidence for an aging deficit in using attention to manage visual WM.

## 1. Introduction

It is widely recognized that healthy older adults show a decline in working memory (WM) compared to younger adults [1,2,3]. WM is a limited-capacity system that maintains a small amount of information available in mind for ongoing cognitive processing. One prominent explanation of WM age-related decline states that older adults have difficulties in using attention to efficiently manage the contents of WM. For example, one may use attention to filter out relevant from irrelevant information, thereby selecting only the relevant information to be encoded to WM [4]. Attention could also be used to ignore irrelevant information (i.e., distractors) presented while WM is engaged in maintaining other information [5,6]. The abilities to filter distractors at encoding (hereafter simply “*filtering*”) and to ignore distractors during memory maintenance (hereafter “*ignoring*”) have been shown to bear different relations to an individual’s WM capacity [7,8]. Furthermore, these abilities can be directed to different objects [9] or different features of an object [10,11].

In the present study, we investigated the hypothesis that age-related cognitive decline in visual WM can be explained by decrease in the ability to filter and ignore distraction. We assessed this hypothesis across two experiments that required participants to attend to different visual objects (Exp. 1) or different features of an object (Exp. 2). In the following sections, we will review the evidence for age differences in these abilities and contextualize our research questions.

### 1.1. Attending to Different Objects

Our visual surroundings are full of visual items: people, animals, and objects. At any given moment, there is too much information for our cognitive system to process. Attention allows us to focus on a subset of these items thereby increasing the processing of relevant and decreasing processing of irrelevant information. For example, if we are looking for our keys, we will ignore the cat and the children running around. If we do not, we might lose focus and forget the places we have already searched.

Attention serves to control which information gets access to WM (for a review see [12]), and studies have shown that the ability to filter and ignore irrelevant information contributes to WM capacity [7,8,13]. Now the question stands: Do deficits in the ability to filter and ignore irrelevant information explain age-related decline in WM? To answer this question, McNab and colleagues [9] used smartphones to assess a sample of over 29,000 people in a game-like task requiring filtering and ignoring of distracting information. In this study, the task consisted in remembering the positions of red circles that appeared on a 4 × 4 grid for 1 s, maintaining them over another 1-s delay, and finally recalling all of the positions by clicking on the respective locations on the grid. Distracting information (yellow circles) could be presented together with the red circles for study thereby requiring the filtering of this information, or they appeared during the delay period, in which case they had to be ignored. Older adults showed larger declines in performance in the presence of distraction than younger adults, but this distraction effect was substantially larger when it occurred during the delay period (thereby requiring ignoring) compared to encoding (requiring filtering). McNab et al. proposed that the greater age-related impairing in ignoring may be explained by the distractors being encoded to WM in the ignore condition by both age groups; however, older adults may have difficulties judging the temporal order of memoranda vs. distractors leading to confusions at recall. There are other alternative explanations though. For example, older adults may require longer postdistraction delays than younger adults to resolve interference due to the slower processing speed associated with aging [14].

In our study, we included conditions to distinguish between these explanations: (a) a condition in which the distractors had to be encoded and maintained in WM instead of ignored (i.e., a high-load baseline) and (b) a condition in which more time was provided to ignore the distractors (ignore+delay condition). The inclusion of a high-load baseline allows one to measure the degree in which performance in the ignore condition reflects encoding of the distractors: if this is the case, performance in the ignore condition should be similar to the high-load baseline. If the distractors are only partially encoded to WM, then performance in the ignore condition should be somewhat better than the high-load baseline. Alternatively, the ignore condition could be even associated with worse performance than the High-Load condition, due to the increased demand of trying to remove distractors and not succeeding leading to loss of relevant items. The inclusion of the ignore+delay condition can clarify whether the difference between the filtering and ignoring conditions is explained by the delayed time-course of distraction exclusion in older age. In the study of McNab et al. [9], there was a 2-s delay between distractor and test in the filtering condition, whereas this time-separation was of only a 1-s in the ignore condition. Several studies have shown that older adults show delayed onset of Event-Related Potential (ERP) markers of distractor-exclusion in filtering tasks compared to younger adults, even in conditions in which equal behavioral performance was obtained between age groups [15,16]. If older adults are slower than younger adults to remove distractors from memory and this explains their worse performance in the ignore condition, then the inclusion of an ignore+delay condition should abolish the age-related decline observed in ignoring.

In sum, older adults seem to have more difficulty ignoring than filtering information, but further research is needed to clarify the reasons for this dissociation. The goal of Experiment 1 was to address the possibilities that this is due to the encoding of the distractors or due to the faulty removal of the distractors from visual WM.

### 1.2. Attending to Different Features

The ability to filter or ignore distractors is assumed to serve visual WM not only when attention is directed to different objects but also when one has to selectively attend to only some of the features of a single object. For example, one may see a large red star and a small blue circle. Remembering the conjunction of multiple features places larger demands on visual WM than when simpler (single-feature) objects are maintained [17,18,19], and this ability is also subjected to large age-related cognitive decline [20,21]. How filtering and ignoring are used when irrelevant information steams from the same (attended) object has been less investigated and, to the best of our knowledge, no study has investigated how it changes in aging.

Research with younger adults have provided mixed answers regarding the question of whether people are able to filter irrelevant features of an object. This question has been examined by comparing conditions in which participants store single-feature objects vs. multifeature objects when (a) only one of the features of the multifeature object is relevant (filtering condition) or (b) all features of the objects are relevant. One of the first studies to address this issue was done by Luck and Vogel [22]. They observed that memory was not affected by the number of visual features within an object, suggesting that participants always encoded all features to visual WM irrespectively of their relevance, and storing more features was not detrimental. More recent studies have provided evidence contradicting these results, showing that storing multiple features consumes more WM capacity [11,17,19,23]. Whether people can filter irrelevant features when they have foreknowledge of which features are relevant has yielded mixed results. Some studies [11,24,25] observed that younger participants could not voluntarily filter irrelevant features, however, other studies have found nearly perfect filtering [26,27,28].

These studies pertain to filtering of irrelevant features at encoding. Information regarding the relevance of some features over others may become available, however, only after the objects were already encoded to WM. A handful of recent studies have also investigated whether people can selectively weight features already encoded to WM, or in other words, whether they can “ignore” irrelevant features in WM [10,29,30]. In these studies, participants encoded a set of multifeatured objects (e.g., colored and oriented gratings). During the retention interval, a cue indicated which feature was relevant for the upcoming test (either color or orientation) or one of the features was tested with no prior warning (no-cue baseline). Compared to the no-cue baseline, cueing the relevant feature during the retention interval yielded better performance.

As reviewed above, only a handful of studies have investigated the ability to filter and ignore features, and these studies were done with younger adults. To the best of our knowledge, no study investigated these abilities in aging. The goal of Experiment 2, therefore, was to assess age-related decline in filtering and ignoring irrelevant features.

### 1.3. The Present Study

Our research questions and hypotheses were preregistered (https://osf.io/jmhwv). As stated in the preregistration, we aimed to investigate age differences in the ability to filter and ignore information in visual WM. In Experiment 1, we investigated the ability to filter and ignore distractor objects. We followed-up on the study of McNab et al. [9], which showed evidence for the greater age-related decline on the ability to ignore distractor objects during maintenance compared to filtering distractors at encoding. In Experiment 1, we tested two hypotheses regarding this age deficit in ignoring. The first possibility is that the sudden onset of the distractors during the retention interval grabs the attention of older adults, leading them to fully or partially encode the distractors to WM. Younger adults, however, can fully ignore the distractors. The second possibility is that both younger and older adults encode the distractors to WM, but younger adults are faster to remove the distractors from WM compared to the older adults. We addressed these possibilities by including conditions that require people to fully encode the distractors to WM (high-load baselines) and conditions that vary the available time to remove distractors from WM (ignore-delay condition).

Experiment 2 extended the investigation of filtering and ignoring abilities to situations pertaining to the encoding and maintenance of multifeature objects. The investigation of the selective weighting of features stored in visual WM is quite recent. Only a few studies have assessed this ability and only among younger adults. Hence, it is not clear whether the ability to ignore irrelevant features would be subjected to more age-related decline than the ability to filter features, similar to the observed for filtering and ignoring of whole objects.

In summary, the current study allows us to investigate four main research issues concerning age-differences in visual WM: (1) Is the inability to ignore distractions in older adults due to their complete or partial encoding? (2) Do older adults take longer to disengage from the information they should ignore? (3) Is there an age difference in the ability to filter irrelevant features in visual WM? and (4) Is there an age difference in the ability to ignore irrelevant features in visual WM?

## 2. Materials and Methods

### 2.1. Participants

Thirty younger students (19–32 years old, *M* = 25.7; 17 women) and thirty older adults (65–79 years old; *M* = 72.6; 14 women) participated in the study. Younger adults were students from the University of Zurich and older adults were Zurich community-dwelling individuals. Participation was compensated with CHF 15 per h or extra course credit (in case of students), and the experimental session lasted between 2 and 3 h. All participants signed the informed consent at the beginning of the experiment. The study protocol was in line with the ethical guidelines of the Institutional Review Board of the University of Zurich and did not require special approval.

All participants confirmed that they have normal or corrected-to-normal visual acuity and color vision. Older adults were assessed with the Mini-Mental Status Exam (MMSE) and all scored higher than 28 (*M* = 29.3).

### 2.2. Materials and Apparatus

Participants were tested in a group laboratory containing four desks, each with a computer. Up to four people could be tested simultaneously. The experimental session was composed of four tasks. The tasks were programmed in MATLAB using the Psychophysics toolbox [31,32]. The experimental tasks proceeded in the following order. First, participants completed two perceptual control tasks measuring their ability to match the color and orientation of visual objects. Next, participants completed the task for Experiment 1 (filter and ignore objects). At the end of this experiment, participants were allowed a 10-min break. Finally, participants completed the task of Experiment 2 (filter and ignore features).

### 2.3. Procedure

#### 2.3.1. Perceptual Matching Tasks

These tasks assessed the motor and perceptual abilities of the participants, and they provided older adults the possibility to practice the continuous adjustment scale used in the memory tasks.

In the color version of the task, two colored dots appeared side-by-side against a grey background (RGB 128 128 128). The dot on the left was colored and served as a target. The dot on the right was dark grey (RGB 90 90 90) and served as the probe. Both dots were surrounded by a color wheel, which was defined in the CIELAB color space with *L* = 70, *a* = 20, *b* = 38, and radius = 60 [33]. The task was to adjust the color of the probe dot to match, as precisely as possible, the color of the target dot. Participants adjusted the probe’s color by moving the mouse around the color wheel, which caused the color of the probe dot to change to the same color as currently selected by the mouse. A mouse-click served to confirm a response, upon which a feedback screen was showed indicating the response of the participant and the correct response.

In the orientation version of the task, the target and the probe were white isosceles triangles presented against a grey background (RGB 128 128 128). Participants had to adjust the orientation of the probe triangle (on the right) to match the orientation of the target triangle (on the left) by moving the mouse around. The tip of the triangle pointed in the same direction as the mouse cursor. As for the other task, a mouse click locked the response and was followed by the presentation of a feedback screen.

Participants completed 4 practice trials and 50 test trials in each task. In both tasks, stimuli were randomly selected from the 360 values of the color wheel or of orientation with the constrain that the selected values for each trial were at least 5° apart from each other. The dependent variable in these tasks was the distance between the reported feature value and the true feature value of the target.

#### 2.3.2. Experiment 1

**Figure 1:**
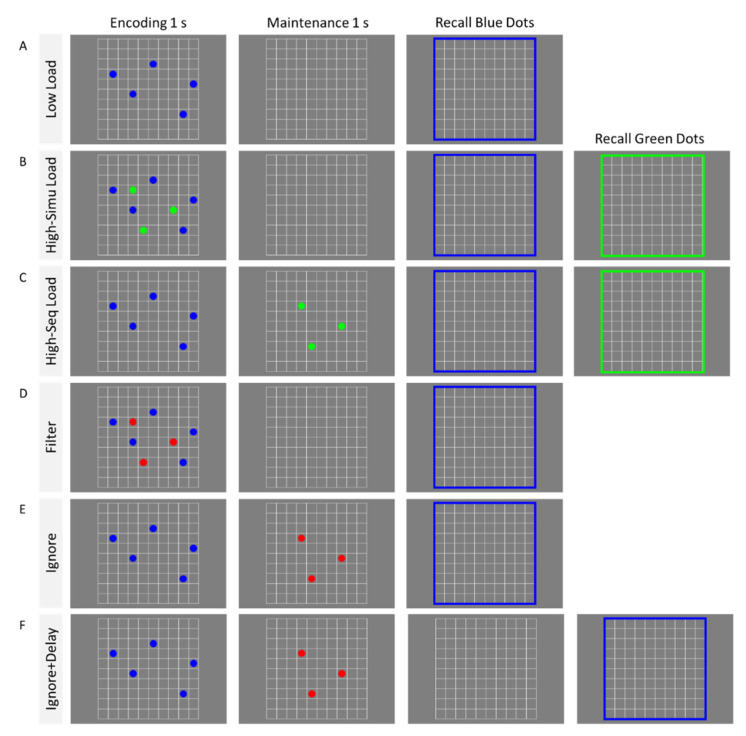
Example of conditions in Experiment 1. In the *Low-Load condition* (**A**) participants encoded blue dots and later reproduced them by clicking on the corresponding grid cells. In *High-Simultaneous Load* (**B**) and *High-Sequential Load* (**C**) conditions, participants encoded both blue and green dots. In the *Filter condition* (**D**), participants encoded the blue dots while ignoring the red dots presented at encoding. In the *Ignore condition* (**E**), participants encoded first the blue dots and ignored the red dots presented during the maintenance phase. In the *Ignore+Delay condition* (**F**) the recall test was presented after an additional delay of 1000 ms.

The experimental task was modeled after the one used by McNab et al. [9]: the position of colored dots on a 10 × 10 grid had to be remembered (see example in Figure 1). The grid was shown in white (RGB 255 255 255) against a uniform grey background (RGB 128 128 128). Participants were told to remember the positions of blue (RGB 0 0 255) and green (RGB 0 255 0) dots, and to ignore any red (RGB 255 0 0) dots that appeared. At test, participants were asked, first, to reconstruct the positions of the blue dots by clicking on the corresponding cells of the grid, followed by reconstruction of the position of the green dots (in case these were presented). The recall of the different types of dots was indicated by the color of the outer frame of the grid. Participants had to click on as many dots as presented in that color before the next recall prompt or the end of the trial was presented. There was no time limit for the recall.

The task was completed across two blocks. In the baseline block, participants were instructed to remember the positions of only blue dots (Low-Load condition) or blue and green dots (High-Load conditions) as detailed below. The number of blue dots was seven for younger and five for older adults. The number of green dots presented in the High-Load conditions was always three for both groups. Different levels of load were presented for each age group because previous research [9] indicated that older adults can store, on average, one-and-half objects less than younger adults do. By reducing the memory load for older adults, we hoped to test the filtering and ignoring abilities at similar levels of difficulty for both age groups. In all conditions, blue dots were tested first, and in the case of the High-Load conditions, this was followed by the test of the green dots. This is because our main question pertains to how well the blue dots can be maintained across all experimental conditions. There were three types of conditions in this block. In the *Low-Load condition* (Figure 1A), only blue dots were showed on the screen for 1000 ms, followed by 1000 ms retention and the recall test. In the *High-Simultaneous Load condition* (Figure 1B), blue and green dots were presented simultaneously on screen for 1000 ms, followed by 1000 ms retention and, lastly, by the recall tests. In the *High-Sequential Load condition* (Figure 1C), blue dots were shown for 1000 ms, followed by the presentation of the green dots for 1000 ms, and then the recall tests. These three conditions serve as the low and high memory-load baseline conditions that allowed us to assess how well participants can filter and ignore distracting information in the main experimental conditions.

In the experimental block, participants were instructed to remember the positions of the blue dots and ignore the red dots. This block again composed of three conditions. In the *Filter condition* (Figure 1D), blue and red dots were presented simultaneously on the screen for 1000 ms, followed by 1000 ms retention, and then the recall test. In the *Ignore condition* (Figure 1E), blue dots were presented first for 1000 ms, followed by the red dots for 1000 ms. The recall test was presented immediately after the offset of the red dots. In *Ignore+Delay condition* (Figure 1F), the recall test was presented after an additional delay of 1000 ms. The inclusion of the postdistraction delay allows us to test for the possibility that older adults need more time to remove the red dots from their WM.

Participants completed 120 trials, divided into the two blocks (whose order was counterbalanced across participants). Given that there were six experimental conditions (i.e., Low-Load, High-Simultaneous Load, High-Sequential Load, Filter, Ignore, and Ignore+Delay), there were 20 trials per design cell. Trials of each condition were randomly intermixed within each block. Six practice trials before each block were done. Opportunities for short breaks were provided every 10 trials.

#### 2.3.3. Experiment 2

**Figure 2:**
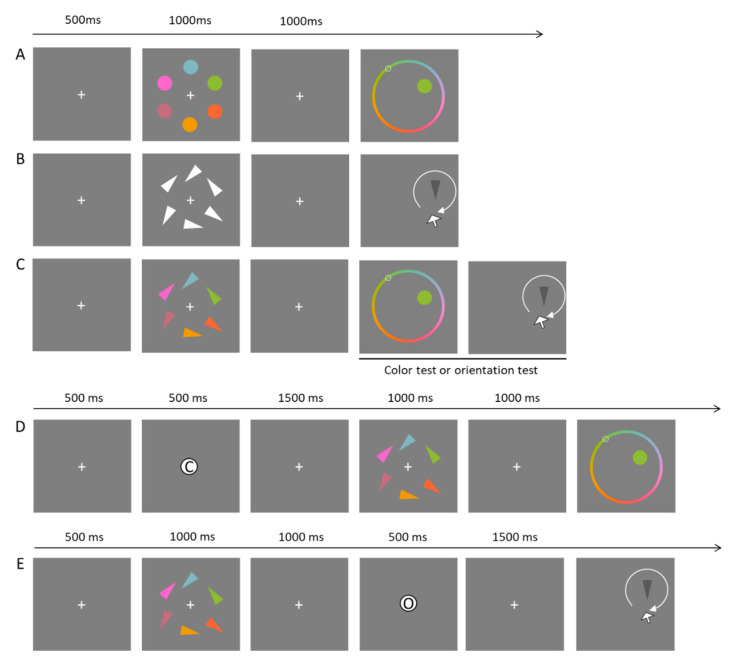
Example of conditions in Experiment 2. In *Single-Feature baseline* conditions, participants encoded only colors (**A**) or the orientation of the triangles (**B**) and then they indicated the color of the target by clicking on the color wheel or the correct triangle’s orientation by moving the mouse to rotate the probe. In *Dual-Feature baseline* (**C**), participants encoded colored triangles, and then either the color or the orientation of an item was probed. In *Filter condition* (**D**), a cue was shown in the middle of the screen indicating the to-be-tested feature (C = color; O = orientation) before the onset of the memory array, allowing participants to only encode the relevant feature to WM. In *Ignore condition* (**E**), a cue was shown during the retention interval, thereby allowing participants to ignore one of the features already stored in WM.

The task in Experiment 2 consisted of remembering visual objects that vary in one or two continuous feature dimensions (i.e., color and orientation), and at test they reproduced one of these dimensions on a continuous scale (see Figure 2).

In the *Single-Feature* baseline conditions, the memory array consisted either of colored dots (which do not contain orientation information; see Figure 2A) or white triangles (which do not contain color information; see Figure 2B). In the test, participants reproduced the single feature of one of the objects in the memory array. In the *Dual-Feature* baseline condition (Figure 2C), participants were presented with colored isosceles triangles, and they remembered both the color and the orientation of these objects. In the test, one object was randomly selected and participants reproduced either its color or its orientation (with equal probability). In the *Filter condition* (see Figure 2D), a precue was presented before the onset of the memory array and indicated with 100% validity whether the color or the orientation of one of the objects would be tested at the end of the trial. The precue allowed participants to only encode the relevant feature to WM. In the *Ignore condition* (see Figure 2E), a retrocue was presented during the retention interval indicating with 100% certainty whether color or orientation would be tested later in the trial. The retrocue allowed participants to ignore, at the test, the uncued feature.

In all conditions, the trial started with a white fixation cross presented against a grey background (RGB 128 128 128) for 500 ms. Next, the memory array (colored dots, white oriented triangles, or colored triangles) was presented for 1000 ms. The memoranda were evenly spaced on an imaginary circle centered on the middle of the screen. Colors were randomly selected from 360 values distributed along with the same color wheel as defined for the color perceptual task. Orientations were selected randomly from 360° angles. After a brief retention interval (1000 ms), memory for one of the stimuli was tested by presenting a dark-grey stimulus (probe) at the location of one of the memoranda. If color was the tested feature, a color wheel was shown. Participants had to move the mouse along the color wheel to adjust the color of the probe to the color of the memory stimulus presented in that location. If the orientation had to be reproduced, only the probe stimulus and the mouse cursor were presented. Participants had to move the mouse around to rotate the probe triangle to match the orientation of the memory triangle that was presented in the same location. Participants pressed the left mouse button to confirm their response. There was no time limit for the recall.

In precue trials (*Filter condition*), a letter (F for color—which is “Farbe” in German and R for orientation—“Richtung” in German) was presented in the middle of the screen for 500 ms indicating which feature (color or orientation) was relevant for the test. The memory array was presented 1500 ms after the offset of the precue. In retrocue trials (*Ignore condition*), the letter F or R was presented 1000 ms after the offset of the memory array. The cue was on screen for 500 ms, and 1500 ms thereafter, the test-array appeared.

The number of objects in the memory array was 6 for younger adults and 4 for older adults. Again, our goal with the presentation of different memory loads between age groups was to present tasks of equivalent difficulty for these groups. Experiment 2 consisted of 400 trials divided into 2 blocks of 200 trials each: a baseline block consisting of the Single- vs. Dual-Feature trials; and an experimental block consisting of the Filter and Ignore trials. There were 4 training trials before each block. Within each block, the type of trial (Dual-Feature vs. Single-Feature; or precue vs. retrocue) was randomized with the constrain that each design cell had an equal number of trials (note that the dual-feature condition had 100 trials, with 50 trials ending in a color test and 50 trials in an orientation test). The order of blocks was counterbalanced across participants. A short break was provided between blocks.

## 3. Data Analysis

The practice trials were excluded from all analyses. In Experiment 1, the dependent variable was the proportion of correctly recalled blue dots across conditions. In Experiment 2, our main dependent variable was the error in reporting the cued feature (either color or orientation) of the tested item across conditions. In the perceptual matching tasks, the dependent variable was the error in matching the feature of the perceptually visible target item. Recall error is the absolute value of the deviation between the true feature value of the tested item and the participants reported feature value.

We analyzed the data using two approaches: *Bayesian model comparison* and *Bayesian parameter estimation*. The first approach compares the likelihood of a stated model given the data. This analysis yields an odds ratio, denominated Bayes factor (BF_10_), which quantifies the evidence for supporting the stated model against the null model. Evidence for the null model over the stated model can be obtained by computing 1/BF_10_ (BF_01_) [34,35]. By comparing models that include different predictors, this procedure allows gathering evidence for or against the presence of main effects and interactions between predictors. Here, we used Bayesian ANOVAs (hereafter BANOVA) and Bayesian *t*-tests to gather evidence for and against our hypotheses.

The second approach computes the relative credibility of all candidate parameter values that can describe the data given the specified model, which yields distributions of the parameter values known as the posterior. To assess the credibility of the parameter, we consider the interval that covers 95% of its posterior (hereafter, the highest density interval–HDI). If the HDI of a parameter does not include 0, or if it is outside of a region around the null, namely, a Region of Practical Equivalence (ROPE; which here was defined around an effect size between −0.1 and 0.1), then the estimated value is considered credibly different from 0. Both analyses were implemented in R [36]: BANOVAs and *t*-tests used the BayesFactor package [37], and parameter estimation used the BEST functions [38].

In our preregistration, we indicated that our main interest in both experiments was in comparing performance in the Filtering and Ignoring conditions concerning both the Low-Load and their respective matched High-Load conditions (High-Simultaneous and High-Sequential, respectively) and to assess age-related change on these. We proposed to compare them using a relative scoring that would combine both comparisons (e.g., (Filtering–High-Simultaneous)/(Low–High-Simultaneous)). However, given the performance levels, we observed in the experiments, this computation yielded uninterpretable values. This happened because some scores in the Filtering and Ignoring conditions were even better than in the Low-Load condition (In our preregistration, we only considered that performance in these distractor conditions would be in-between the Low-Load condition and the High-Load condition. It turns out that performance tended to be even better in these conditions than in the Low-Load baseline). Therefore, we decided to report separate comparisons with each of these load baselines to allow for proper testing of our stated hypotheses. For these comparisons, we used a two-tailed *t*-test. For comparisons between age-groups, however, we used a one-tailed *t*-test to more specifically test the hypothesis that older adults perform more poorly than younger adults.

## 4. Results

The study materials, data, and analysis scripts are available at the Open Science Framework at https://osf.io/ezr3m/.

### 4.1. Experiment 1

For one participant in the younger group, data of 10 trials was lost due to a computer crash.

**Figure 3:**
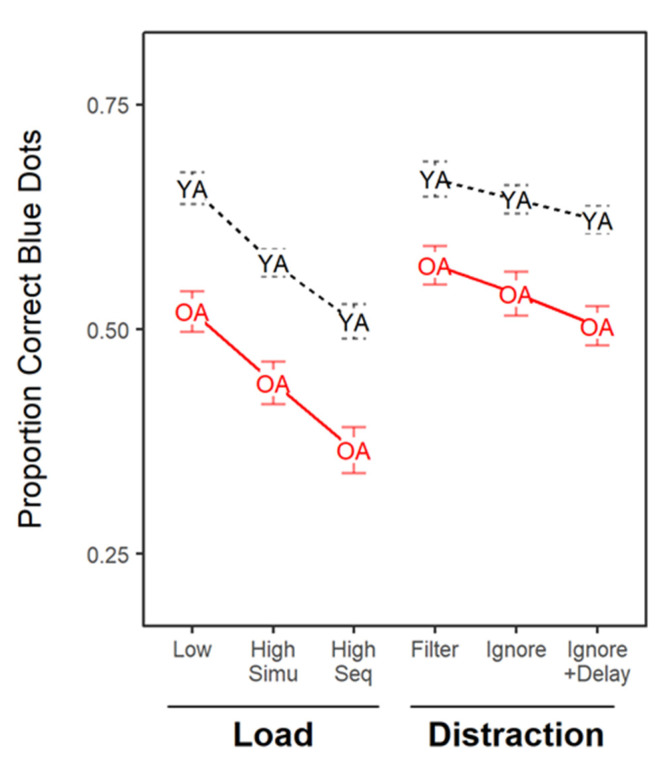
Proportion of correctly recalled blue dots in Experiment 1 for each age group (YA = younger adults, OA = older adults) across the experimental conditions that varied in memory load (Low, High-Simultaneous, and High-Sequential) and distractor presence (Filter, Ignore, and Ignore+Delay).

Figure 3 shows performance in Experiment 1 as a function of the experimental condition for the two age groups. Overall, younger adults performed better in the task than older adults, even though we provided younger participants with a higher memory load (7 blue items) than older adults (5 blue items). Figure 3 also shows that there is no sign of interactions between experimental condition and age. Accordingly, a BANOVA including age (younger vs. old) and condition (Low, High-Simu, High-Seq, Filter Ignore, and Ignore+Delay) showed that the best model of this data included only the main effects of condition and age (BF_10_ = 2.90 × 10^58^). This model was substantially preferred to the model including the interaction term (BF_10_ = 0.27). There was substantial evidence to include the main effects of age (BF_10_ = 3.56 × 10^53^) and condition (BF_10_ = 6.93 × 10^4^) in the best model. We also assessed age effect only on the conditions involving distraction (Filter, Ignore, and Ignore+Delay). The best model of this data only included the main effects of age and condition (BF_10_ = 2.96 × 10^9^), and there was substantial evidence to exclude the interaction term (BF_10_ = 0.17). These results indicate that although older adults were able to recall fewer positions correctly, the performance of older and younger adults was similarly affected by memory load and distraction presence.

#### 4.1.1. Filtering Ability

Table 1 shows contrasts between the *Filter* condition and the *Low-* and *High-Load* conditions, respectively. As indicated in Table 1, the *Filter* condition tended to yield better performance than the *Low-Load* condition, and this improvement was credible for older adults. This result is the opposite of what is predicted by a deficit in filtering irrelevant objects. We tested for the directional hypothesis that this filtering score was lower for the older adults than for the younger adults with a one-sided *t*-test, which our results show clear evidence against.

The comparison against the *High-Simultaneous* condition indicated that the *Filter* condition produced better performance in both age groups. We tested for an age difference in filtering against this baseline assuming that older adults performed worse than younger, but our results showed strong evidence against this difference. If anything, the older adults had a larger filtering benefit than the younger adults.

#### 4.1.2. Ignoring Ability

Table 1 also presents the contrast between the *Ignore* condition and the *Low-* and *High-Load* conditions, respectively. The contrast of the *Ignore* condition to the *Low-Load* condition indicated the absence of credible changes in performance for both younger and older adults. Critically, there was substantial evidence against age differences, indicating that older adults were as able as younger adults to ignore irrelevant objects.

Comparison to the *High-Sequential Load* condition revealed better performance in the *Ignore* condition for both older and younger adults. This outcome indicates that the older adults did not encode the items that they should ignore; indeed, encoding additional items overloads WM capacity leading to worse performance in the *High-Sequential* condition, which was not the case for the *Ignore* condition. Again, there was no evidence for an age-related difference in ignoring ability.

#### 4.1.3. Delay Effect: More Time to Ignore Distractors?

Our last question was whether providing a delay after the presentation of the distractors could improve the ignoring of the distractors. Our data showed, unexpectedly, that neither younger nor older adults had problems ignoring the distractors as presented in the previous section. Indeed, as shown in Table 1, the presentation of a longer delay after the distractors was associated with worse performance in the *Ignore*+*Delay* condition compared to the *Ignore* condition. There was no age-related difference in this effect.

### 4.2. Perceptual Matching Tasks

Before Experiment 2, participants completed two perceptual matching tasks that allowed us to assess their motor and perceptual abilities with regards to the reproduction of colors and orientations. Younger adults (*M* = 2.80, *SD* = 0.85) tended to reproduced the colors with smaller error compared to older adults (*M* = 3.19, *SD* = 1.33), but this difference was not credible, BF_10_ = 0.5, with 14% of the credible effect size (*M* = −0.32) values within the ROPE (i.e., effect size between −0.1 and 0.1). For orientation reproduction, younger adults (*M* = 3.90, *SD* = 2.70) showed similar performance to older adults (*M* = 3.57, *SD* = 1.64), and for this comparison, the null hypothesis was preferred by a factor of about 3, BF_10_ = 0.30, with evidence of 25% of the credible effect size values (*M* = 0.06) within the ROPE. These results indicate that any differences in performance between younger and older adults in terms of memory performance in Experiment 2 cannot be attributed to worse perceptual-motor abilities.

### 4.3. Experiment 2

**Figure 4:**
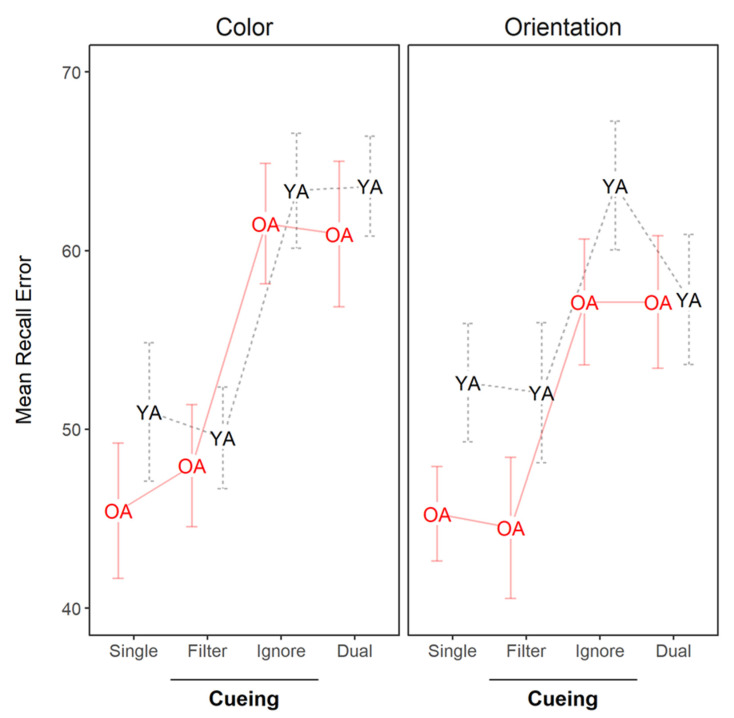
Mean recall error in Experiment 2 for each age group (YA = younger adults, OA = older adults), recall (color and orientation), and *baseline condition* (Single and Dual) and *cueing condition* (Filter and Ignore).

Figure 4 presents the recall error for reproducing the colors and orientations in Experiment 2. Overall, performance was best when participants encoded single-feature objects (colored dots or oriented triangles) and worse when they encoded dual-feature objects (colored triangles). The *Filter* conditions yielded performance similar to the *Single-Feature* conditions. The *Ignore* conditions, in contrast, yielded performance similar to the *Dual-Feature* conditions.

We ran a BANOVA on these data having conditions (Single, Filter, Ignore, Dual), recalled feature (color vs. orientation), and age group (younger vs. older) as fixed predictors, and participant as a random effect. The best model included only the main effect of condition (BF_10_ = 1.42 × 10^31^). The evidence against including the main effect of the recalled feature was ambiguous (BF_10_ = 0.70) as well as of including the main effect of age group (BF_10_ = 0.63). These results suggest that performance in Experiment 2 mainly varied with the condition.

To facilitate gathering evidence for age differences, we also ran two separate BANOVAs, one for recall of colors and one for recall of orientations. For color recall, the best model of the data only included the main effect of condition (BF_10_ = 1.76 × 10^22^). There was ambiguous evidence against excluding the main effect of age group (BF_10_ = 0.48). Critically, there was strong evidence against including the interaction of age group and condition (BF_10_ = 0.10). For orientation recall, the best model of the data also only included the main effect of condition (BF_10_ = 9.99 × 10^11^). There was ambiguous evidence against excluding the main effect of age group (BF_10_ = 0.78) and for excluding the age group × condition interaction (BF_10_ = 0.74). Notice that this is probably because the younger group tended to perform worse in the *Ignore* condition than the *Dual-Feature* condition.

As there was no credible main effect of recalled feature in the BANOVA (presented above), the analyses of the main effects of Bayesian Estimation (Table 2) were calculated from the average performance across recall of color and orientation.

#### 4.3.1. Filtering Ability

Table 2 presents the comparison of the *Filter* condition against the *Single-Feature* and *Dual-Feature* conditions. The contrast of *Filter* vs. *Single-Feature* provided evidence against credible differences for both age groups, and there was also substantial evidence against an age reduction in filtering efficiency. This is pattern of results expected given perfect filtering of the irrelevant feature at encoding.

The contrast of *Filter* vs. *Dual-Feature* showed smaller recall error in the *Filter* condition. There was substantial evidence that storing both features yielded worse recall error for both age groups (i.e., *Single* vs. *Dual* contrast). Older adults tended to show a larger cost in the Dual-Feature condition, but this effect was not credible.

Overall, the results are consistent with the hypothesis that feature filtering efficiency was unaffected by age.

#### 4.3.2. Ignoring Ability

Table 2 presents the comparison of the *Ignore* condition against the *Single-Feature* and *Dual-Feature* conditions. The contrast *Ignore* vs. *Single-Feature* showed larger recall error in *Ignore* condition for both age groups, indicating that participants were unable to ignore the irrelevant feature in WM.

The contrast *Ignore* vs. *Dual-Feature condition* showed worse recall in the *Ignore condition,* particularly for the younger group. There was, however, no credible evidence for an age effect on ignoring ability.

## 5. General Discussion

The current study investigated age-related differences in the efficiency of using attention to manage the contents of WM, specifically in filtering and ignoring irrelevant objects (Exp. 1) and in filtering and ignoring irrelevant features of the object (Exp. 2).

Our experiments revealed the following main findings: older adults were as able as younger adults to efficiently filter and ignore distractor objects (Exp. 1). Furthermore, older and younger adults performed similarly in filtering distractor features (Exp. 2). This experiment also showed no evidence that younger and older adults could ignore a feature they already encoded to visual WM. Overall, Experiment 2 also pointed to a lack of evidence that older adults have deficits in managing the contents of visual WM in comparison to younger adults.

Next, we will discuss the implications of these findings in terms of attention to objects and features.

### 5.1. Attentional Modulation of Objects

McNab and colleagues [9] showed evidence that filtering of irrelevant objects would be relatively preserved in aging, but ignoring irrelevant objects would be impaired. Here, we were interested in investigating whether the inability to ignore distractions in older age was due to the full or partial encoding of the distractors. The results provide evidence of efficient ignoring of distractor objects in both age groups. Although we set up to test explanations of an ignoring deficit in aging [9], we failed to observe an age-related cognitive decline in ignoring as revealed by three findings: (1) there was no interaction between age and distractor condition, indicating that both age groups were similarly affected by the presence of distractors at encoding and during the delay; (2) comparison of performance in the Filter and Ignore conditions against our Low-Load baseline showed no evidence of a drop in performance, and (3) the better performance observed in the Filter and Ignore conditions compared to the High-Load baselines provided evidence against the hypothesis that distractors were encoded to WM.

Our results stand in opposition to the results from the study of McNab et al. [9]. There are several differences between our experiments that may explain these discrepant findings, which we will discuss below.

The first refers to the task setup. Our experiment involved a larger grid of spatial locations (10 × 10) than used by McNab et al. (4 × 4). We decided against a small grid because in such a grid, participants can use the strategy to remember the empty cells instead of the filled ones. Our decision to increase the grid was also related to the fact that in the study of McNab et al., younger adults were at ceiling performance in their baseline (no-distraction) condition, whereas this was not the case for the older adults. This potentially precluded them from properly measuring the impact of distraction in the younger group, because the impact of ignoring was assessed relative to this baseline.

A second difference between our studies refers to the memory load between age groups. Based on the McNab et al.’s data, we estimated that older adults could store, on average, 1.5 items less than younger adults, so we provided a load of 2-items less to the older adults in an attempt to keep task difficulty at a similar level between age groups. We note that this load difference cannot explain age differences in Experiment 1. On the one hand, although increasing memory load improves the chance of getting a response correctly, our grid had 100 locations, and hence chance probabilities were of 5% and 7% for older and younger adults, respectively. This gives younger adults an advantage of 2% over older adults; nonetheless, age differences between conditions were of, at least, 10% in average as shown in Table 1. On the other hand, we relied on a within-subjects comparison to determine the efficiency of filtering and ignoring, and for this comparison, guessing probability is constant. Despite this load manipulation, our results showed that older adults still performed more poorly overall than younger adults, suggesting that age differences might have been underestimated in McNab et al. due to the ceiling performance of younger adults. Critically, the memory load selected for the younger adults (7 items) was effective to bring performance of the younger adults below ceiling, permitting us to attest that the younger participants were performing at their maximal WM capacity even at our Low-Load (no-distraction) baseline.

A third difference refers to the fact that our Low-Load condition was randomly intermixed with the High-Load conditions. Arguably, the Low-Load (no-distraction) condition may produce even better performance if participants are not exposed to a situation in which they are constantly required to process the color of the dots in order to determine their category (blue vs. green). This requirement may have forced participants to remember not only the spatial locations of the dots, but also their color. Maintenance of color-location bindings is arguably more demanding than maintenance of only locations. This could have created a more difficult baseline than the one used by McNab et al., which did not involve this additional requirement. This may have led our study to underestimate the impact of distraction in the Filter and Ignore conditions. Future studies are needed to properly disentangle whether there are costs associated with Filtering and Ignoring when these conditions are contrasted to a baseline in which only location needs to be remembered vs. a baseline in which color-location bindings may need to be remembered. This will help to establish whether the costs of distraction are associated with the need to form bindings when distraction is presented. That said, we believe this does not undermine our main finding that age was not associated with impairments in ignoring information, because we still did not observe an interaction between age and distraction (Filter vs. Ignore), a contrast that is not confounded by baseline comparisons.

A fourth difference is that McNab et al. ran a span procedure, whereas our experiment consisted of a fixed memory load for several trials. In a span procedure, participants are exposed to trials with increasing memory load (in their study this ranged from 2 to 10 items). If the participant responds correctly in a number of trials (e.g., 2) with a given load, the load is increased in the next trial by one unit. When participants make a mistake, the trial is repeated, and if they repeatedly fail to recall all items correctly, the task ends. The last memory load in which perfect recall was observed is defined as the memory span of the participant. In such a procedure, memory span is measured within a couple of trials. This procedure, however, is probably disadvantageous for older adults because they are not provided enough trials to develop a proper strategy to manage the task. Younger adults might be faster in figuring out the best strategy to deal with the increasing memory load giving them an advantage in this type of procedure.

Overall, our study supports the conclusion that older adults are not impaired relative to younger adults in ignoring distractor objects when we provide them with a sufficiently large number of trials with the task and guarantee that younger and older adults were performing at maximal capacity.

In Experiment 1, we also wanted to determine whether older adults needed more time to ignore irrelevant objects. Aging is well-known to be associated with a decline in cognitive processing speed [39]. This age-related slowing also influences WM processes, e.g., the time required to encode a stimulus [40,41]. Contrary to our expectations, our attempt to provide more time to ignore the distractors in the *Ignore+Delay* condition did not help performance. Instead, performance decrements were observed indicating that there was time-based forgetting of the spatial representations of the to-be-remembered items due to the prolongation of the retention interval [42]. Importantly, this was observed for both age groups.

Regarding the ability to filter distractors, our findings replicate previous results that showed similar filtering performance between younger and older adults [9,16]. This suggests that older adults can maintain similar performance levels compared to their younger counterparts. Whether the mechanisms associated with this preserved performance are the same between age groups is still unclear. Some neurophysiological studies have observed age-related impairment in the degree of activity suppression in areas processing distractor information [40] and on the timing of onset of ERPs associated with suppression of distractors [16]. Together, these results suggest that older adults might need more time to efficiently deploy attention, but otherwise have intact attentional processing abilities, and hence, they can also manage their WM contents efficiently.

### 5.2. Attentional Modulation of Features

Experiment 2 examined the ability to selectively attend to only some of the features of a single object during encoding (also known as filtering) and during the maintenance period (also known as ignoring). Our results demonstrated similar performance in the *Filter* and *Single-Feature* conditions and strong evidence for better performance in the *Filter* than the *Dual-Feature* condition in both age groups. These results are in line with the hypothesis that participants used the precue to only encode the relevant feature to visual WM, efficiently filtering the irrelevant feature dimension. Encoding of this feature yielded a cost to performance as demonstrated by the *Dual-Feature* condition and replicating other studies showing WM capacity limitations in storing multifeature objects [17,18]. The evidence of a filtering feature benefit provides support for the initial encoding process that can be biased by feature-based selective attention [43,44]. Selectively encoding of relevant features can provide advantages to WM performance. Relevant features may be encoded faster and even more robustly in visual WM. This may also make the retrieval process less costly, considering that the more features are encoded, the more decision processes will be needed, increasing the probability of errors [44]. Critically, there was no age difference in filtering irrelevant features: younger and older adults were equally able to use the precue to select only the relevant feature for encoding into visual WM.

To determine whether it is possible to ignore a feature after it has been encoded into visual WM, we presented a retrocue during the retention interval indicating the relevant feature for the test. Performance in the *Ignore* condition was similar to the one in the *Dual-Feature* condition, indicating that the multifeature object was maintained intact until tested, and the irrelevant feature could not be ignored. If feature-based attention could modulate the selection of a single feature for recall, then we would expect similar performance in the *Ignore* and *Single-Feature* conditions or at least better performance than in the *Dual-Feature* condition, which our data clearly shows evidence against.

There are not many studies addressing the ability to ignore irrelevant features. Two previous studies compared a Dual-Feature condition to a feature retrocue condition and reported feature retrocue benefits [10,29]. It is unclear why we could not replicate these results here. We can rule out the possibility that the participants did not understand the meaning of the cue: the same cues were used in the *Filter* and the *Ignore* conditions, which were randomly intermixed across trials. Participants were extremely efficient in using the cue if it appeared before the memory array, thereby yielding filtering benefits, but not if it appeared after the memory array, which did not produce an ignoring effect. We can only speculate on why feature retrocue benefits were not observed. One difference that could be relevant is that studies obtaining feature retrocue benefits had a lower memory load (2 or 3 items) than used here (6 items) with younger participants, and somewhat longer postcue times (2000–5000 ms), whereas in our study, this delay was of 1500 ms. Future studies are needed to understand under which conditions participants are able to use feature-based attention to prioritize relevant features of objects already stored in visual WM.

In summary, Experiment 2 provided evidence against the hypothesis that older adults have a deficit in directing feature-based attention to representations in visual WM compared to younger adults.

### 5.3. Aging Deficits in the Control of Attention?

A deficit in using visual attention to control visual WM contents has been proposed as one of the main contender explanations for visual WM impairments in aging [45]. This hypothesis predicts that older adults exhausts their WM capacity with irrelevant information, thereby performing more poorly for tests of the relevant information. What is the evidence supporting this hypothesis? Some neurophysiological studies have pointed to an age-related deficit in selective attention as revealed by comparison of brain activations vs. deactivations in face of requirements to attend vs. filter/ignore information [4,46,47,48] or time for onset of suppression markers in ERP components [16]. Behaviorally, however, the evidence is more challenging for the inhibition hypothesis. Selective attention is beneficial for encoding and maintaining relevant content in visual WM as revealed by precue and retrocue benefits, respectively (for a review see [49]). Studies investigating age-related deficits in the use of selective attention through attentional cues provide challenging results to this inhibition-deficit hypothesis. Some studies showed no age-related deficits in precue performance [50,51], which is akin to the filtering ability investigated in the present study (Exp. 1) and in the study of McNab et al. [9]. In conjunction, these studies and ours show that older adults do not have difficulties in focusing their attention in perceptually available relevant visual information, thereby gating them access to visual WM, and hindering access to distractor information. These results resonate with findings questioning age-related deficits in executive control more generally, which use visual perceptual tasks coupled with distraction manipulations, such as Stroop, Flanker, or Simon tasks [52,53,54]. This literature has revealed that patterns of age-related decline can be fully explained by reductions in processing speed. Results from retrocue paradigms, conversely, are akin to the use of attention to ignore an irrelevant content maintained in visual WM [55]. Although some initial studies pointed to age-deficits in using retrocues [56,57], subsequent studies have consistently obtained retrocue benefits of similar magnitude between younger and older adults [50,58,59,60,61]. Together with the present findings, more and more behavioral evidence indicates that the source of the robust age-related decline observed in visual WM is unlikely to be explained by deficits in inhibiting irrelevant information.

### 5.4. Limitations

In the present study, we only investigated the contribution of visual attention to visual WM performance. Our results therefore may not generalize to verbal working memory tasks, which may involve different brain areas and different mechanisms to gate and maintain information in mind [62].

Another limitation is that we only investigated healthy aging. Filtering and ignoring distractors in visual WM may be impaired in nonhealthy aging (e.g., dementia) in addition to the visual WM deficits that have been found in several neurodegenerative diseases [63,64]. Our results may serve as a benchmark, however, when assessing the impact of nonhealthy changes that can occur in aging.

Lastly, our sample size (*N* = 30 per group) was modest, and our age range limited to elderly between 65 and 79 years old (*M* = 72.6), hence not covering old-old age. Future studies may include larger sample sizes and broad age ranges to more fully understand the impact of aging on the relation between attention and visual WM.

## 6. Conclusions

The current study investigated age-related differences in filtering and ignoring irrelevant objects and irrelevant features of an object. Although inefficient control of attention is usually assumed to underlie age differences in WM capacity, across two experiments, we found no support for the assumption that the older adults are less able than younger adults to filter and ignore distraction in visual WM when selecting different objects or features within an object. Hence, their lower visual WM capacity cannot be explained by the inefficient use of attention to control the contents of visual WM.

## Figures and Tables

**Table 1:** Analysis of the data of Experiment 1. Mean recall error and 95% highest density interval (HDI) estimated from Bayesian *t*-tests assessing (1) the age effect in each condition, (2) the filter, ignore, and delay effect within each age group, and (3) the age effect on the size of the filter, ignore, and delay effects.

	*Age Group*				*Age Effect (Older < Younger)*					
	Younger		Older		Raw Score		Effect Size			
**Condition**	M	95% HDI	M	95% HDI	M	95% HDI	M	95% HDI	ROPE	BF_10_
Low	0.66	[0.62,0.70]	0.52	[0.49,0.56]	0.14	[0.09,0.19]	1.47	[0.85,2.10]	0	5 × 10^4^
High-Simu	0.57	[0.54,0.61]	0.44	[0.40,0.48]	0.13	[0.08,0.18]	1.38	[0.82,2.05]	0	3.4 × 10^4^
High-Seq	0.51	[0.48,0.54]	0.36	[0.33,0.40]	0.15	[0.10,0.19]	1.57	[0.92,2.28]	0	1.4 × 10^5^
Filter	0.67	[0.63,0.71]	0.58	[0.54,0.61]	0.10	[0.05,0.15]	1.03	[0.45,1.60]	0	162
Ignore	0.64	[0.61,0.68]	0.54	[0.51,0.58]	0.10	[0.06,0.15]	1.10	[0.55,1.73]	0	970
Ignore+Delay	0.62	[0.59,0.66]	0.50	[0.47,0.54]	0.12	[0.07,0.17]	1.33	[0.71,1.89]	0	4500
**Contrasts**										
***Filter = Low-Load***										
Raw score	0.01	[−0.02,0.04]	0.05	[0.02,0.08]	−0.04	[−0.08,0.0]	−0.50	[−1.07,0.0]	0.05	0.09
Effect size	0.14	[−0.24,0.53]	0.70	[0.28,1.12]						
*p*(ROPE)	0.31		0							
BF_10_	0.25		57							
***Filter = High-Simu***										
Raw score	0.09	[0.07,0.12]	0.13	[0.10,0.17]	−0.04	[−0.08,0.0]	−0.48	[−1.04,0.06]	0.07	0.10
Effect size	1.52	[0.96,2.09]	1.38	[0.85,2.09]						
*p*(ROPE)	0		0							
BF_10_	3.9 × 10^6^		5.3 × 10^5^							
***Ignore = Low-Load***										
Raw score	−0.01	[−0.04,0.01]	0.02	[−0.01,0.05]	−0.03	[−0.07,0.0]	−0.41	[−0.96,0.11]	0.09	0.11
Effect size	−0.18	[−0.58,0.19]	0.22	[−0.14,0.62]						
*p*(ROPE)	0.25		0.20							
BF_10_	0.32		0.39							
***Ignore = High-Seq***										
Raw score	0.14	[0.11,0.17]	0.18	[0.13,0.22]	−0.04	[−0.09,0.01]	−0.44	[−0.95,0.13]	0.09	0.11
Effect size	1.83	[1.23,2.55]	1.66	[1.05,2.27]						
*p*(ROPE)	0		0							
BF_10_	1.7 × 10^8^		1.5 × 10^7^						
***Ignore-Delay > Ignore***										
Raw score	−0.02	[−0.04,0.00]	−0.04	[−0.06,−0.01]	0.01	[−0.02,0.05]	0.14	[−0.33,0.71]	0.23	0.16
Effect size	−0.41	[−0.82,−0.02]	−0.48	[−0.90,−0.10]						
*p*(ROPE)	0.05		0.02							
BF_10_	0.07		0.06							

Note: Simu = Simultaneous; Seq = Sequential. For each effect, the evidence (BF) for the alternative hypothesis over the null is presented (BF_10_) for a one-sided test. ROPE = probability of values within a region of practical equivalence (effect size between –0.1 and 0.1). For within-subject condition comparisons (e.g., Filter = Low-Load), we performed two-tailed *t*-tests. For between-subject comparisons (e.g., older < younger), we performed a one-tailed *t*-test.

**Table 2:** Analysis of recall error in Experiment 2. Mean recall error and 95% highest density interval (HDI) estimated from Bayesian *t*-tests assessing (1) the age effect in each condition, (2) the load (single/dual), filter, and ignore benefit in each age group, and (3) the age effect on the condition effect.

	*Age Group*				*Age Effect (Younger < Older)*					
	Younger		Older		Raw Score		Effect Size			
**Condition**	**M**	**95% HDI**	**M**	**95% HDI**	**M**	**95% HDI**	**M**	**95% HDI**	**ROPE**	**BF_10_**
Single-Feature	51.8	[46.9,56.8]	45.0	[39.8,49.8]	6.84	[−0.15,13.7]	0.51	[−0.02,1.06]	0.05	0.10
Color	50.8	[45.4,56.2]	45.2	[39.7,50.3]	5.66	[−1.81,13.3]	0.40	[−0.14,0.93]	0.10	0.11
Orientation	52.8	[47.0,58.5]	44.9	[39.4,50.4]	7.92	[0.06,16.0]	0.52	[−0.02,1.10]	0.05	0.10
Dual-Feature	60.6	[55.7,65.5]	59.1	[54.8,63.5]	1.54	[−4.81,7.95]	0.07	[−0.39,0.67]	0.26	0.20
Color	63.7	[58.9,68.3]	60.9	[57.0,65.0]	2.73	[−3.31,8.83]	0.26	[−0.28,0.77]	0.20	0.15
Orientation	57.4	[51.4,63.6]	57.3	[51.2,63.2]	0.12	[−8.7,8.23]	−0.02	[−0.52,0.52]	0.30	0.26
Filter	50.7	[44.1,57.1]	45.6	[40.0,51.0]	5.06	[−3.79,13.2]	0.32	[−0.22,0.85]	0.15	0.14
Color	49.4	[43.4,55.4]	47.4	[42.0,52.5]	1.98	[−5.93,9.94]	0.14	[−0.41,0.66]	0.25	0.20
Orientation	52.1	[44.7,59.8]	44.0	[37.0,51.0]	8.07	[−2.02,18.5]	0.37	[−0.12,0.95]	0.09	0.12
Ignore	63.4	[58.6,68.6]	58.9	[54.6,63.6]	4.47	[−2.04,11.4]	0.35	[−0.19,0.89]	0.13	0.13
Color	63.2	[58.1,68.4]	61.0	[56.0,65.6]	2.22	[−5.11,9.1]	0.19	[−0.37,0.72]	0.23	0.18
Orientation	63.8	[57.9,70.0]	56.8	[51.2,62.6]	7.00	[−1.10,15.3]	0.45	[−0.09,1.01]	0.07	0.11
**Contrasts**										
***Dual > Single***										
Raw score	8.51	[4.91,12.1]	13.7	[9.45,18.0]	−5.15	[−10.7,0.42]	−0.47	[−1.04,0.03]	0.06	2.29
Effect size	0.90	[0.47,1.37]	1.21	[0.74,1.76]						
*p*(ROPE)	0		0							
BF_10_	2402		1.6 × 10^5^							
***Single = Filter***										
Raw score	1.2	[−2.93,5.17]	−0.80	[−4.09,2.55]	2.01	[−3.04,7.40]	0.19	[−0.33,0.73]	0.22	0.16
Effect size	0.11	[−0.27,0.49]	−0.11	[−0.48,0.28]						
*p*(ROPE)	0.34		0.36							
BF_10_	0.22		0.22							
***Dual = Filter***										
Raw score	9.92	[6.40,12.10]	13.2	[8.93,17.40]	−3.20	[−8.57,2.24]	−0.32	[−0.86,0.21]	0.15	0.80
Effect size	1.08	[0.58,1.70]	1.17	[0.66,1.85]						
*p*(ROPE)	0		0							
BF_10_	4776		1.7 × 10^5^							
***Single = Ignore***										
Raw score	−11.4	[−14.8,−8.02]	−14.0	[−17.7,−10.6]	2.57	[−2.19,7.41]	0.33	[−0.24,0.84]	0.18	0.64
Effect size	−1.35	[−1.91,−0.84]	−1.49	[−2.17,−0.94]						
*p*(ROPE)	0		0							
BF_10_	4.9 × 10^5^		2.5 × 10^6^							
***Dual = Ignore***										
Raw score	−3.14	[−6.34,0.17]	−0.22	[−3.54,3.03]	−2.88	[−7.57,1.72]	−0.32	[−0.86,0.20]	0.14	0.87
Effect size	−0.40	[−0.78,0.02]	−0.00	[−0.40,0.35]						
*p*(ROPE)	0.08		0.40							
BF_10_	0.97		0.19							

Note: For each effect, the evidence (BF) for the alternative hypothesis over the null is presented (BF_10_) for a one-sided test. ROPE = probability of values within a region of practical equivalence (effect size between –0.1 and 0.1). For most within-subject condition comparisons (e.g., Single = Filter), we performed two-tailed *t*-tests, except for the comparison of Dual > Single, for which a substantial body of research creates the expectation of worse performance in the Dual than the Single condition. For between-subject comparisons (e.g., younger < older), we performed a one-tailed *t*-test.
